# Cross-sectional study of the association between empathy and burnout and drug prescribing quality in primary care

**DOI:** 10.1017/S1463423619000793

**Published:** 2019-10-30

**Authors:** O Yuguero, JR Marsal, M Esquerda, L Galvan, J Soler-González

**Affiliations:** 1 Transversal Emergency Research Group, Biomedical Research Institute of Lleida (IRBLLEIDA), Lleida, Spain; 2 Surgery Department Faculty of Medicine, University of Lleida, Lleida, Spain; 3 CIBER of Epidemiology and Public Health (CIBERESP), Barcelona, Spain; 4 Epidemiology Unit of the Cardiology Department, University Vall d’Hebron Hospital, Barcelona, Spain; 5 Unitat de Suport a la Recerca Lleida, Institut Universitari d’Investigació en Atenció Primària Jordi Gol (IDIAP Jordi Gol), Lleida, Spain; 6 Borja Bioethics Institute, Barcelona, Spain; 7 Pharmacy Department, Catalan Health Service, Lleida, Spain

**Keywords:** burnout, empathy, ethics, pharmacy, prescription, primary health care

## Abstract

**Objective:**

The aim of this study was to assess whether burnout and empathy levels among general practitioners (GPs) might influence prescribing performance assessed using pharmaceutical prescription quality standard indicators.

**Design and Setting:**

Cross-sectional descriptive study of 108 GPs from 22 primary care centers in Lleida, Spain, and of centralized data corresponding to 183 600 patients under their care. The study was conducted between May and July 2014.

**Main Outcome Measures:**

Burnout and empathy were measured using the Spanish versions of the Maslach Burnout Inventory and the Jefferson Scale for Physician Empathy, and prescribing quality was measured using the Catalan Pharmaceutical Prescription Quality Standard (EQPF). Normal distribution of scores was verified using the Chi-square and Kolmogorov–Smirnov–Lilliefors tests. The effect of each of the variables was evaluated using crude odds ratios.

**Results:**

Older GPs scored significantly higher in the EQPF (*P* < 0.05). High empathy scores were positively associated with high EQPF scores. GPs with low burnout also performed better in the EQPF.

**Conclusions:**

More empathic, less burned-out, older GPs showed better prescribing performance according to quality indicators. However, further studies are needed to evaluate other factors influencing prescribing habits. The promotion of communication skills may increase empathy and reduce burnout, thus benefiting patients.

## Introduction

Quality standards are being increasingly used to analyze the functioning of different health care services, including primary care (Choi, 2016). In Catalonia, an autonomous region of Spain, two quality indicator systems are used to assess prescribing practices in relation to the appropriate use of drugs: the Pharmaceutical Prescription Quality Index (IQF) (Catalan Ministry of health, 2016), created in 1999, and the Pharmaceutical Prescription Quality Standard (EQPF) (Catalan Ministry of Health, 2017), created in 2003.

The EQPF was designed to incentivize better prescription of drugs in primary care practice. The quality criteria and indicators are supported by scientific evidence (Gilabert-Perramon, 2010) and are designed for multidisciplinary teams with the aim of increasing the use of the most cost-effective drugs and ultimately reducing the enormous variability associated with the treatment of the most common diseases dealt with in primary care.

Choice of treatment can logically influence the course or outcome of a disease (Cebrià, 2003). The same applies to treatment adherence, which has been directly linked to doctor–patient communication (Kerasidou and Horn, 2016). Several studies (Yuguero *et al*., 2012) have shown that patient-oriented attitudes and communication skills play an important role in improving adherence to treatment and bringing about lifestyle changes. Other studies, in turn, have evaluated the impact of the work climate and prescribing practices on quality of care (De Dios, 2007; Pandraud-Riguet, 2017; Yuguero *et al*., 2017b).

Empathy, which is the ability to understand another person’s feelings and thoughts and to communicate that understanding (Hojat *et al*., 2002b), has been described as a modulator of doctor–patient communication. Empathic engagement by health care professionals has been linked to multiple benefits for the patient, including better communication (Zachariae, 2003) and higher levels of satisfaction and treatment adherence (Kelley *et al*., 2014). In Spain, resident programs in family and community medicine and nursing include specific training (with defined objectives) in professionalism, communication skills, and empathy (Spanish Ministry of Health, 2005).

Physician burnout (Maslach *et al*., 2001) can also affect doctor–patient communication (Medscape, 2017) and, consequently, quality of care (Shanafelt *et al*., 2015), and it appears to be a growing problem as physicians and other health care professionals are increasingly exposed to greater workloads and social pressure (Shanafelt, 2009). Burnout has also been described in medical students (Brazeau *et al*., 2010) and residents (Leiter *et al*., 2013; Kwah *et al*., 2016).

The burnout syndrome described by Maslach is measured in three dimensions: emotional exhaustion, personal accomplishment, and depersonalization (Maslach and Jackson, 1981). Burned-out physicians show greater fatigue and cynicism (Portoghese *et al*., 2014), as well as a poorer understanding of their patients’ needs (West *et al*., 2018), and this can have a negative impact on communication and patient management (Colville, 2018).

Our group has undertaken several studies to analyze the impact of burnout and empathy among general practitioners (GPs) and nurses on different aspects of primary care practice (Yuguero *et al*., 2017a). Moreover, this relation between empathy and burnout has also been described in other countries (Sun *et al*., 2017; Pedersen *et al*., 2018) and in other health care groups among emergency professionals (Yuguero *et al*., 2017a), psychiatrists (Bentley *et al*., 2018) and intensive care professionals (van Mol *et al*., 2015). In all cases, high empathy has been described as a contributing factor of burnout prevention.

The principal goal of the present study was to investigate the association between levels of burnout and empathy among GPs and prescribing practices based on the EQPF pharmaceutical prescription quality standard. The secondary objectives are to describe this association according to gender, age, and place of work. Until now, studies linked to empathy and burnout of health professionals have been oriented toward clinical problems (West *et al*., 2018), but we believe that we must look further and seek the implications in management. Our main hypothesis is that more empathic professionals, with less burnout, prescribe better. This is in reference to prescribing drugs when the patient requires them and following the scientific recommendations, and not influenced by the economic interests of the pharmaceutical industry. We also consider whether there are any differences in terms of gender or age. Regarding age, we take into account that in our country, many training activities for younger people are financed by the pharmaceutical industry and a prescription may be expected in exchange.

## Materials and methods

### Participants and study design

We conducted a descriptive study with volunteer participants from the Catalan health care region of Lleida, which has 22 primary care centers serving a population of approximately 366 000 people. All GPs working at these centers (205 people) were contacted by email and asked to complete surveys between May and July 2014 on burnout and empathy. The overall response rate was 52.6%. In this study, we analyze the answers provided by 108 GPs with 183 600 patients under their care. All results were anonymized to ensure confidentiality.

### Instruments and variables

#### Burnout evaluation

Degree of burnout was measured using the Spanish version of the Maslach Burnout Inventory (MBI). This version has been validated (Maslach and Jackson, 1987; Gil-Monte, 2005), and used in previous studies (Yuguero *et al*., 2015; Fernández-Sanchez *et al*., 2017). The MBI is used to measure stress and burnout in a work environment and contains 22 items scored on a 7-point Likert scale on which responses range from never (score of 0) to every day (score of 6). The MBI includes three subscales: emotional exhaustion (MBI-EE), depersonalization (MBI-DP), and personal accomplishment (MBI-PA). Higher scores for MBI-EE and MBI-DP indicate higher levels of burnout. The opposite, however, is true for MBI-PA, where lower scores are associated with higher burnout (Álvarez Gallego and Fernández Ríos, 2001).

The GPs were classified into low, medium, or high burnout groups based on their scores on the three subscales and the MBI as a whole. The cutoff scores used for the three subscales were as follows: <22 (low), 22–31 (medium), and >31 (high) for MBI-EE; <7 (low), 7–13 (medium), and >13 (high) for MBI-DP; and <30 (low), 30–35 (medium), and >35 (high) for MBI-PA. These are the cutoffs indicated by the authors of the original questionnaire.

#### Empathy evaluation

Empathy was measured using the Spanish version of the Jefferson Scale for Physician Empathy (JSPE)(Alcorta-Garza *et al*., 2005), a validated 20-item scale recognized as the gold standard for measuring medical empathy (Hojat *et al*., 2002a). An example item from the scale is ‘My patients feel better when I understand their feelings’. In the JSPE, respondents indicate how strongly they agree with each item on a scale of 1–7. Higher scores indicate greater empathy.

The GPs were also divided into three groups according to their level of empathy (low, medium, or high), but in this case there were no pre-established cutoffs. The average empathy score is considered to lie around 120 (Hojat *et al*., 2002a) points and we followed previous strategies of classifying empathy levels as high for mean scores plus 2 SDs and as low for mean scores minus 2 SDs.

#### Evaluation of drug prescribing quality

The EQPF has two sections: an overall section that very general assesses quality criteria (such as the use of new drugs), and a specific section that assesses aspects related to drug prioritization and use for certain diseases. Each section contains indirect indicators obtained from billing data for drugs dispensed by the Catalan Government (CatSalut).

For each indicator, a numerical target is scored based on the values of the best positioned GPs in all Catalonia. That is, based on the score obtained by professionals who prescribe more appropriately, the scores for evaluating all professionals are established. The achievement of each target is scored and weighted according to its relevance and difficulty. The scores for each section are added up and the total possible score for the area of family and community medicine ranges from 0 to 100. Table 1 shows the full list of indicators used in the EQPF.

**Table 1.** EQPF pharmaceutical prescription quality standard indicators

Indicators^a^	Target^b^	Score (points)^c^
Global		
% of drugs not recommended by the Catalan Health Institute or with better therapeutic options	≤1% ≤1.2% ≤1.5% >1.5%	15 11 7 0
Specific		
*Use of antihypertensive drugs* % of recommended diuretics % of ARBs/(ACE inhibitors + ARBs) % of recommended antihypertensive drugs	≥30% ≥26% ≤32% ≤37% ≤43% ≥70% ≥64%	2 1 4 3 2 4 3
*Use of antiulcer drugs* % of recommended PPIs/total PPIs	≥92% ≥90%	8 4
*Use of drugs for musculoskeletal pain* % of recommended NSAIDs/total anti-inflammatory drugs	≥70% ≥65%	8 4
*Use of drugs for osteoporosis* % of recommended osteoporosis drugs	≥60% ≥50%	8 4
*Use of antibiotics* % penicillin/total antibiotics % amoxicillin/(amoxicillin + clavulanic acid) % of recommended antibiotics Population exposure rate	≥60% ≥50% ≥74% ≥70% ≤7 ≤9	1 1 2 1 4 2
*Use of hypolipidemic drugs* % of recommended hypolipidemic drugs	≥84% ≥81% ≥77%	10 7 4
*Use of drugs for respiratory diseases* % of long-acting beta2 agonists + corticosteroids % of recommended COPD/ASTHMA drugs	≤25% ≤30% ≥87% ≥82%	3 1 5 3
*Use of antidepressants* % of recommended antidepressants	≥65% ≥61%	8 4
*Use of hypnotics* % of recommended hypnotics	≥89% ≥87%	8 4
*Use of antidiabetic drugs (non-insulin)* % of drugs recommended	≥85% ≥82% ≥78%	9 6 3
Maximum score		100

a Indicators column shows the global indicator for the whole EQPF, and specific indicators are related with drug prescription in specific diseases established by the Catalan Health Institute (ICS), using percentage of recommend drugs in each case.

b This column refers to the target established by the ICS to evaluate each indicator. For each indicator there are different options of achievement, that is, in the global indicator, the ICS has established as the gold standard to prescribe less than 1% of non-recommended drugs. However, professionals can obtain less than 1.2% or less than 1.5% of non-recommended drug prescriptions.

c Depending on their achievement, they will obtain different scores. If the minimum score is not achieved, 0 points are obtained. Scores obtained depend on achievement.

EQPF = Pharmaceutical Prescription Quality Standard; ARBs = angiotensin II receptor blockers; ACE = angiotensin-converting enzyme; PPIs = proton-pump inhibitors; NSAIDs = nonsteroidal anti-inflammatory drugs; COPD = chronic obstructive pulmonary disease.

The EQPF was created to evaluate the quality of prescribing practices in primary care (Amado *et al*., 2000) and is endorsed by the Pharmacy Commission of the Catalan Health Institute (ICS). It is subject to continuous review and its criteria and results have contributed to the creation of common Drug Therapeutic Guidelines for the entire ICS. Pharmacological prescription is electronic in Catalonia, and most prescriptions are linked to a single GP, facilitating the study of different links between patients and specific GPs (Gilabert-Perramon, 2010).

We recorded individual EQPF scores for 2014 obtained by the GPs who completed the burnout and empathy surveys.

#### Other variables

The following sociodemographic data were recorded: age, sex, and place of work classified as urban (center located in the capital) or rural.

### Data analysis

The initial analysis consisted of a descriptive study of the categorical variables and the results obtained for the MBI, JSPE, and EQPF. The reliability of the instruments was tested by calculating Cronbach’s *α*, which was 0.733 for the MBI and 0.748 for the JSPE. The Chi-square and Kolmogorov–Smirnov–Lilliefors tests were used to check the normal distribution of the questionnaire scores. The distributions of the variables are not distributed as a normal distribution, so we decided to use a nonparametric test. In order to analyze the association between the sociodemographic variables and JSPE, MBI, and EQPF scores, the results were grouped into three categories (low, medium, and high) following a previously described system (Yuguero *et al*., 2017b). Categorical data were described using absolute and relative frequencies. Numerical data were described using means and standard deviation. The Kruskal–Wallis or the Mann–Whitney nonparametric test was used depending on the number of categories. The effect of empathy (JSPE) and burnout (MBI) in the EQPF was assessed using linear regression while the effect of the other characteristics on empathy was assessed using logistic regression. Since our sample consists of volunteer doctors, there may be a certain selection bias. Our hypothesis is that the physicians evaluated could achieve a better empathy score, but we do not dare to give our opinion in the case of burnout. As the work guaranteed total anonymity, we cannot perform qualitative work on those professionals in order to look more deeply into the reasons that lead our professionals to prescribe differently. We have carried out different statistical analyses in order to evaluate the presence of other factors that may explain the results. However, we have not detected the presence of other confounding factors.

Also, a correlation analysis of Spearman’s Rho was performed and a linear regression model was adjusted. Results are shown in Table 2.

**Table 2.** Correlation and linear effect between EQPF and MBI and JSPE

Correlation	Spearman’s Rho	*P*
MBI	−0.072	0.474
JSPE	0.153	0.128
Linear model	Crude effect (CI95%)	*P*
MBI	−0.102 (−0.332; 0.129)	0.384
JSPE	0.345 (−0.053; 0.743)	0.088

EQPF = Pharmaceutical Prescription Quality Standard; MBI = Maslach Burnout Inventory; JSPE = Jefferson Scale for Physician Empathy

The size of the effect in Table 3 has been assessed using the crude OR with a 95% confidence interval, and relative risk (95%CI) in Tables 4 and 5.

**Table 3.** Professional burnout according to GPs’ empathy levels

Empathy (JSPE)	Medium–Low (*n* = 65)	High (*n* = 43)	Total (*n* = 108)	*P*	OR (95% CI)
	*N* (%)	*N* (%)	*N* (%)		
Total burnout (MBI)				0.005	
Mean (SD)	39.3 (20.5)	31.5 (18.3)	36.2 (19.9)	0.042	0.98 [0.96–1.00]
Low	29 (44.62%)	31 (72.09%)	60 (55.56%)		Ref.1
Medium	30 (46.15%)	11 (25.58%)	41 (37.96%)		0.34 [0.15–0.81]
High	6 (9.23%)	1 (2.33%)	1 (2.33%)		0.16 [0.02–1.37]
Emotional exhaustion (MBI-EE)				0.166	
Low	33 (50.77%)	26 (60.47%)	59 (54.63%)		Alcorta-Garza et al. (2005)
Medium	9 (13.85%)	8 (18.6%)	17 (15.74%)		1.13 [0.38–3.33]
High	23 (35.38%)	9 (20.93%)	32 (29.63%)		0.5 [0.2–1.25]
Depersonalization (MBI-DP)				0.037	
Low	32 (49.23%)	33 (76.74%)	65 (60.19%)		Alcorta-Garza et al. (2005)
Medium	19 (29.23%)	3 (6.98%)	22 (20.37%)		0.15 [0.04–0.57]
High	14 (21.54%)	7 (16.28%)	21 (19.44%)		0.48 [0.17–1.36]
Personal accomplishment (MBI-PA)				<0.001	
Low	10 (15.38%)	2 (4.65%)	12 (11.11%)		Alcorta-Garza et al. (2005)
Medium	33 (50.77%)	9 (20.93%)	42 (38.89%)		1.36 [0.25–7.37]
High	22 (33.85%)	32 (74.42%)	54 (50%)		7.27 [1.45–36.47]

JSPE = Jefferson Scale for Physician Empathy; MBI = Maslach Burnout Inventory

**Table 4.** Drug prescription according to GP empathy levels

Empathy (JSPE)	Medium–Low	High	Total	*P*	RR (95% CI)
	Mean (SD)	Mean (SD)	Mean (SD)		
Number of criteria with 100% accomplishment	3.39 (2.6)	3.26 (2.5)	3.34 (2.5)	0.785	1 (0.9–1.1)
Hypertension					
Percentage of diuretics	25.28 (6.8)	28.06 (6.4)	26.34 (6.8)	0.044	1.1 (1–1.1)
percentage of angiotensin II receptor blockers	37.04 (10.1)	33.53 (11.3)	35.71 (10.6)	0.133	1 (0.9–1)
percentage of recommended antihypertensive drugs	64.29 (8.2)	68.14 (9.2)	65.75 (8.8)	0.041	1.1 (1–1.1)
Dyslipemia					
Percentage of recommended hypolipidemic drugs	78.5 (9.1)	80.77 (9.2)	79.36 (9.2)	0.223	1 (1–1.1)
Diabetes Mellitus					
Percentage of recommended antidiabetic drugs	81.38 (10.3)	85.02 (7.8)	82.76 (9.6)	0.060	1 (1–1.1)

JSPE = Jefferson Scale for Physician Empathy

**Table 5.** Drug prescription according to GP burnout levels

Burnout (MBI)	Low	Medium–High	Total	*P*	RR (95% CI)
	mean (SD)	mean (SD)	mean (SD)		
Number of criteria with 100% accomplishment	3.7 (2.7)	2.9 (2.2)	3.3 (2.5)	**0.031**	0.87 [0.8–0.98]
Hypertension					
Percentage of diuretics	27.4 (7)	25.1 (6.4)	26.3 (6.8)	0.065	0.95 [0.9–1.01]
Percentage of angiotensin II receptor blockers	35.5 (10.1)	35.9 (11.4)	35.7 (10.6)	0.981	1 [1–1.04]
Percentage of recommended antihypertensive drugs	66.4 (7.9)	65 (9.7)	65.8 (8.8)	0.633	0.98 [0.9–1.03]
Dyslipemia					
Percentage of recommended hypolipidemic drugs	79.5 (9.7)	79.3 (8.6)	79.4 (9.2)	0.790	1 [1–1.04]
Diabetes Mellitus					
Percentage of recommended antidiabetic drugs	83.3 (9.9)	82.2 (9.2)	82.8 (9.6)	0.374	0.99 [0.9–1.03]

MBI = Maslach Burnout Inventory

All results are presented with 95% CIs. All analyses were performed using the SPSS (v. 15) and statistical significance was established at *P* < 0.05.

## Results

### Quality of prescription and sociodemographic variables

We obtained data on 108 GPs. The sample consisted of 69 women (63.9%) and the mean age was 49.2 years. Of these professionals, 54.65% are working in rural areas. The mean EQPF score was 55.2 points, with older GPs scoring significantly higher (*P* < 0.05). GPs with a high EQPF score had a mean age of 49.8 years, while those with a low score had a mean age of 46.8 years. No significant associations were observed between EQPF scores and gender or place of work.

The results for the association between empathy and burnout scores are shown in Table 3. High empathy is associated with low burnout (*P* < 0.05). There are no significant differences in relation with gender, age, or place of work.

### Empathy and quality of prescription

Empathy scores were high for 43 GPs (39.9%) and medium or low for 65 (60.1%). GPs with high empathy scored better in the EQPF, but the differences were not significant (*P* = 0.072). In addition, there was a positive association between high empathy and a high EQPF score (OR = 1.02). Neither the correlation analysis nor the linear regression model displayed any statistically significant differences. Spearman’s correlation, in terms of burnout score, reveals that in addition it scores lower in the EQPF but not significantly (*P* = 0.474). Regarding the burnout scale, it is observed that for each point increase in the MBI scale, the EQPF descends on average 0.102 points (not significantly).

**Figure 1.**
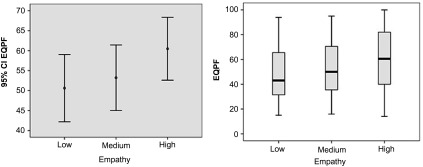
EQPF results according to GPs’ empathy levels. Trends observed for prescribing quality according to empathy. Professionals with high empathy obtain better results in the EQPF.

Figure 1 shows the trends for prescribing quality as a function of empathy, with an evident upward trend. Professionals with high empathy obtain higher scores in the EQPF.

The EQPF can be disaggregated by diagnoses. Three indicators, for example, are used to assess prescribing quality for hypertensive patients. The indicators used for diabetes and dyslipidemia are percentages of recommended drugs for these disorders. As can be seen in Table 4, compared with GPs with low or moderate empathy, GPs with high empathy scored higher in percentage of diuretics and percentage of recommended hypertension treatments but lower in percentage of angiotensin receptor blockers.

### Burnout and quality of prescription

Just over half of the GPs (55.56%) had low burnout. There appeared to be an association, albeit without statistical significance, between burnout and prescribing quality, as the lower the level of burnout, the higher the EQPF score.

The statistical analysis of correlation and regression also shows that higher empathy increases the EQPF score (though not significantly) (see Table 2). It is also determined that a one-point increase in the empathy scale implies an increase of 0.345 points in the EQPF (almost significantly *P* = 0.088) (see Table 2).

**Figure 2.**
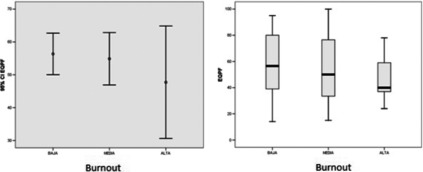
EQPF results according to GPs’ burnout levels. Trends observed for prescribing quality according to burnout. Professionals with moderate burnout obtain higher results.

The trend for prescribing quality was less clear than empathy as a function of burnout and is shown in Figure 2. In this case, it can be seen that professionals with high burnout obtain a lower EQPF score. However, professionals with low burnout also obtain low scores.

**Figure 3.**
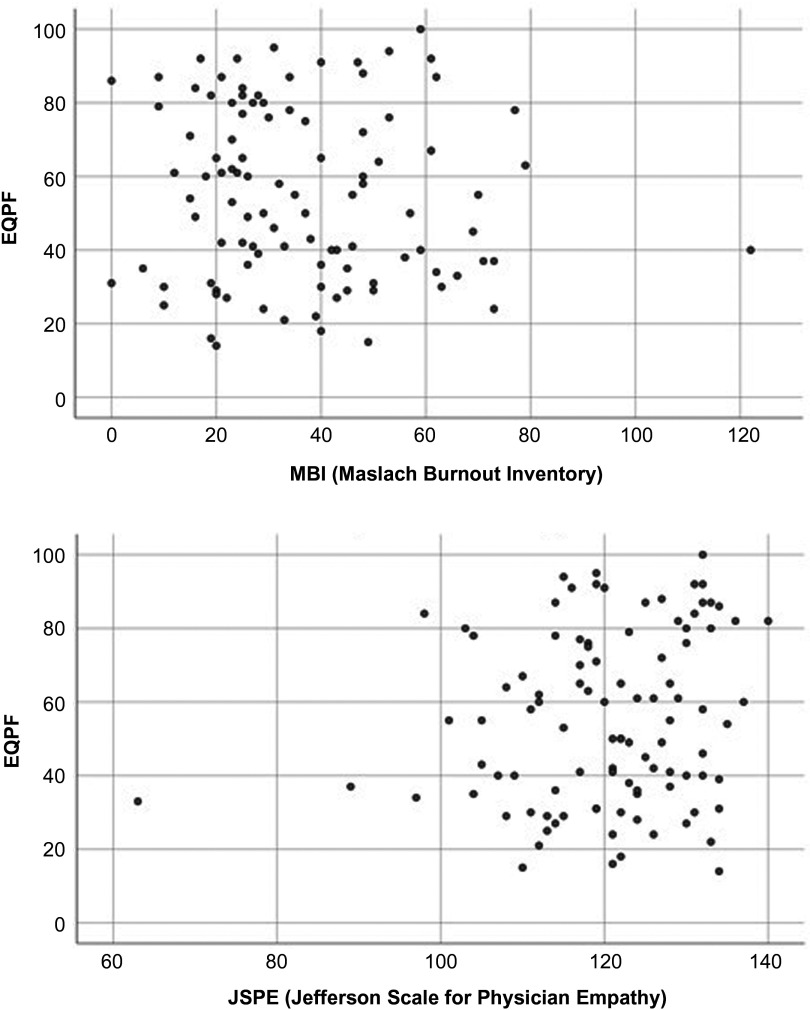
Spearman’s correlation between Maslach Burnout Inventory Scale, Jefferson Scale of Empathy and EQPF results.

Figure 3 shows very visually the correlation of the two scales.

In relation with cardiovascular prescriptions, Table 5 shows that physicians with low burnout scored higher in percentage of diuretics and percentage of recommended hypertension treatments. Statistically significant differences were observed for percentage of diuretics (to treat hypertension) but not for percentage of diabetes drugs.

## Discussion

### Summary

Our findings show that prescribing practices assessed using international quality indicators were significantly better among more empathic and older GPs. A nonsignificant trend toward higher quality prescribing was also noted for GPs with low burnout.

### Strengths and limitations

One potential limitation of our study is our relatively small sample size. In future studies, it would be interesting to survey more GPs, including those from other health districts. This would improve the power of our study, possibly enabling us to detect significant results for both empathy and burnout. To guarantee the professionals’ anonymity, we cannot establish a qualitative method to ascertain the reasons for prescribing differently. We plan qualitative studies in the future to approach this issue in an open, multicausal perspective.

The main limitation of this study is its cross-sectional design, which prevents any investigation of causal associations. Qualitative studies to investigate the trends detected would also be very useful, as mentioned above. Another limitation of our study is related to our grouping of MBI and Jefferson scores into two categories (Low and Medium–High for burnout and High and Medium–Low for empathy), as this could affect the validity of our results. Finally, our analysis of prescribing practices was limited to three cardiovascular diseases that are particularly prevalent in our community, but we believe that similar analyses with other prevalent chronic diseases covered by the EQPF are warranted.

We believe that our findings are important. Considering the current economic situation in which health care expenditure is tightly controlled, it is important to better understand the different factors that influence prescribing practices. More empathic GPs may be more aware of the importance of rationalizing the use of drugs, while less burned-out GPs might be more motivated to help relieve strains on the health care system. This study may lead other research teams to investigate the economic situation of public health and the physicians’ implication. Finally, greater efforts should be made to favor empathic engagement and communication skills among primary care workers and to improve working and social conditions to relieve burnout.

Although one of the strengths of the work is to explore the relationship of drug prescription with empathy and burnout, it is important to note that there are many more related factors. For this reason, we believe it is very important to continue investigating all the factors that are associated with the prescription process, and to do so from different perspectives and adding other scales in the analysis, such as the Scale of Work Engagement and Burnout (Hultell and Gustavsson, 2010). It might also be useful to explore the differences in relation to the types of drugs prescribed.

### Comparison with the existing literature

The EQPF is an important indicator of the quality of health care in Catalonia. Like the healthcare quality standard (EQA) (Coma *et al*., 2013), a synthetic indicator of quality of care, it is based on indicators used in the UK health system and is revised regularly. These indicators are assessed annually to monitor the performance of health care centers and staff. All drugs prescribed by GPs in the Catalan health care system are recorded electronically and available for analysis. However, despite the potential value of assessing the quality of prescribing practices in primary care (Christensen *et al*., 2016), few studies have used these indicators.

When GPs in the Catalan health system prescribe a drug that does not meet the established suitability criteria, they receive confirmation to this effect. In addition, they can consult their performance through the centralized computer system. Apart from helping GPs to achieve the targets established by the Catalan Institute of Health, these indicators are part of a pay-for-performance system, which would presumably also affect prescribing practices beyond levels of empathy or burnout (Vila *et al*., 2005). A study of GPs in Barcelona in 2003 found that higher levels of burnout were associated with greater pharmaceutical expenditure (Cebrià, 2003). This is the same year in which the EQPF was established in Catalonia.

Our findings show a significant association between empathy and prescribing quality. Empathically engaged GPs obtained higher EQPF scores, and in addition, they performed significantly better when it came to prescribing recommended diuretics for the control of hypertension and antihypertensives overall. We found no significant associations for the prescription of drugs recommended for dyslipidemia and diabetes. We chose to focus on hypertension, dyslipidemia, and diabetes to facilitate comparisons with other studies (Hojat, 2011) and because they are highly prevalent in primary care and are characterized by considerable variability in prescribing practices and the constant appearance of new drugs. One recent study of primary care practice in the United States found no significant associations between prescribing practices for acute respiratory infections and empathy or burnout (Pedersen *et al*., 2018).

Although we did not detect any statistically significant relationship between burnout and prescribing quality, we did observe a tendency toward better results among less burned-out professionals. Based on reports for Spanish GPs in 2007 (Yuguero *et al*., 2018), this might be explained by their greater commitment to expenditure control and the sustainability of the health system (Orton and Pereira Gray, 2016). However, more studies are needed to investigate these trends and evaluate other factors that could affect prescribing practices.

Older GPs scored better in the EQPF. Their performance in this respect could be influenced by their greater experience, but it is also possible that they prescribe more drugs that are not penalized, perhaps because they prefer to continue working with drugs that have provided them with good results. In short, prescribing practices were better among more empathic and older GPs, and this ultimately benefits the patient (Melo *et al*., 2016).

Our sample, despite being small, is representative of our region and also of our territory. Despite this, we believe that our results can be generalized to other primary care teams. The working conditions are similar throughout our territory, and we believe that it should be up to the managers of the health institutions to promote communication skills.

### Implications for research and practice

Future lines of research could investigate ways to achieve improvements in the above areas, since training and techniques to enhance empathy and reduce burnout among health care professionals could improve pharmaceutical expenditure, quality of care, health outcomes, and patient quality of life. In conclusion, we believe that health institutions should be aware of the factors influencing professionals’ drug prescription and treatment adherence. By investing in promoting empathy, benefit can be obtained by reducing burnout but also involving professionals in prescription expenditure.

### Ethical aspects

The study was approved by the Primary Care Research Institute (IDIAP) Clinical Research Ethics Committee. Data confidentiality and anonymity were ensured in accordance with the Spanish Data Protection Law 15/1999. All data were encoded and accessible only to the primary care information system technician who cross-referenced the data. Because the database was anonymous, the researchers were at no time able to identify the study participants.
